# Household Demography and Early Childhood Mortality in a Rice-Farming Village in Northern Laos

**DOI:** 10.1371/journal.pone.0119191

**Published:** 2015-03-16

**Authors:** Shinsuke Tomita, Daniel M. Parker, Julia A. Jennings, James Wood

**Affiliations:** 1 Graduate School of Agriculture, Kyoto University, Kyoto, Japan; 2 Department of Anthropology and Population Research Institute, Pennsylvania State University, University Park, Pennsylvania, United States of America; 3 Department of Anthropology, University at Albany, State University of New York, Albany, New York, United States of America; University of Washington, UNITED STATES

## Abstract

This paper extends Alexandr Chayanov’s model of changing household demography (specifically the ratio of food consumers to food producers) and its influence on agricultural behavior so that it includes possible adverse effects of a rising ratio on nutritional status and early childhood mortality within the household. We apply the model to 35 years’ worth of longitudinal demographic and economic data collected in the irrigated-rice growing village of Na Savang in northern Laos. When appropriate controls are included for other household variables, unobserved inter-household heterogeneity, and changes in local conditions and national policy over the study period, the analysis suggests that a unit increase in the household’s consumer/producer ratio induces something like a nine-fold increase in the risk of death among household members aged less than five years. Monte Carlo simulation studies suggest that this may be an over-estimate but also that the effect is probably real and likely to be an important factor in household demography. At the very least, the results suggest that Chayanov’s model still has theoretical relevance and deserves to be revived.

## Introduction

Between 1912 and 1920, the Russian agronomist Alexandr Vasil’evich Chayanov [1–2] developed a simple model of the effect of demographic processes on the food-producing behavior of subsistence-oriented, economically self-sufficient farming households. Although Chayanov limited his attention to peasants, drawing from his own field studies among grain-growing peasant farmers in the Ukraine’s “black earth” region, his model has long since been generalized to other preindustrial farmers as well [3–4]. The basic idea of the model was straightforward, even if its application to reality has often proved to be problematic. The present paper is intended to resolve some of the model’s major problems and to apply an extended version of it to empirical data on early childhood mortality among irrigated-rice farming villagers in the mountains of northern Laos.

### Chayanov’s model and its limitations

Every farming household, Chayanov noted, undergoes a kind of *demographic life cycle* associated with birth, death, and out-marriage, as a result of which the household goes through distinct phases of formation, expansion, contraction, and extinction or dissolution. (Chayanov’s treatment of this life cycle was, by his own admission, extremely schematic. More realistic household models can be found in the recent demographic literature; for reviews, see [5–6].) According to Chayanov, the number and age- and sex-composition of household members change throughout this cycle, and to understand the economic implications of these changes it is important to take account of the fact that not all of its members are equivalent. Infants need to be fed but do not contribute to the food supply; even older children who are involved to some degree in working the farm may not be meeting all their own nutritional needs, let alone those of the larger household. Men and women have different physiological work capacities as well as different nutritional requirements, both complicated in women by the additional burdens of pregnancy and lactation. Older members may have declining work capacities, but they still need to eat. Thus, raw numbers on household size by themselves are unlikely to be very informative. Instead, the individual-level data on the household members must be weighted by age- and sex-specific coefficients that reflect changes in both the members’ productive capacities and their nutritional requirements over the course of their life spans.

This weighting scheme provides, for each household at any one time, a weighted number of food producers (P) and a separate weighted number of food consumers (C). Both C and P change over the household life cycle, but because the age- and sex-specific weights are not the same for C and P (e.g. infants eat but do not produce), they change at different rates. As a result, the ratio between the two, C/P, itself changes over the household’s life cycle and is likely to be an important determinant of how hard the farmers have to work to meet their households’ nutritional needs—assuming that farmers seek to avoid unnecessary “drudgery” (*tyagostnost*) whenever possible and thus need to be pressured to work harder. In other words, Chayanov identified possible demographic inducements to agricultural intensification operating strictly at the level of the household rather than that of the whole population.

The translation of some of Chayanov’s major works into English for the first time in the mid-1960s [8] inspired a considerable body of empirical research intended to test the idea that household C/P ratios exert an important effect on the intensity of household food production in traditional rural economies [4], [9–10]. After two decades of research, however, the results of these tests turned out to be equivocal: sometimes the results were positive, sometimes negative, and sometimes too obscure to provide a clear resolution of the question [3–4], [11–20]. On the basis of these apparently contradictory results, most researchers appear to have concluded that Chayanov was either wrong or that the effects of C/P ratios he postulated are trivial or too variable across populations to allow any useful generalizations to be made about them.

We suggest that the failure to resolve the question about the impact of C/P ratios in empirical research stemmed from several problems, both statistical and conceptual, that affected all studies of Chayanov’s ideas, regardless of results. At a *minimum* the difficulties include those listed in Table 1. Given these analytical problems, it is reasonable to conclude that Chayanov’s model was never really given a fair hearing in the older literature. Because it is a simple model (and we would argue that this is a virtue), it is necessary to control for other confounding variables statistically in order to test it adequately. Earlier studies simply did not attempt to do this, and their small sample sizes would have precluded it anyway.

**Table 1:** Conceptual and statistical problems affecting most earlier tests of Chayanov’s model of C/P ratios, including Chayanov’s own.

Small samples (generally < 50 households, often approx. 10–15)
Simple bivariate analyses—thus, no statistical control of confounding influences
Almost purely cross-sectional data (although the model is about prospective changes in household composition and subsistence behavior)—thus, no allowance for time-varying effects
Poor measures of productive intensity (P)
• Mostly focused on a single staple crop
• Measured as hours of work as opposed to energy expended
• Too restrictive a definition of work—esp., no allowance for domestic work^a^
Poor measures of consumption (C)
• Also tend to be measured on one or a very few staple crops
• Usually measured from total household yield on the assumption (almost certainly wrong) that each member is allocated food proportional to his/her nutritional needs as read from a published table of international dietary standard
• Do not include breastfeeding, either as a food source for the child or as a metabolic burden for the mother
No attention to C/P effects on the *demand* side of the household economy (the weighted number of mouths to be fed) as opposed to the supply side (the size of the household food supply)

^a^Except in [11], which justly criticizes other studies for this shortcoming.

**Fig 1:**
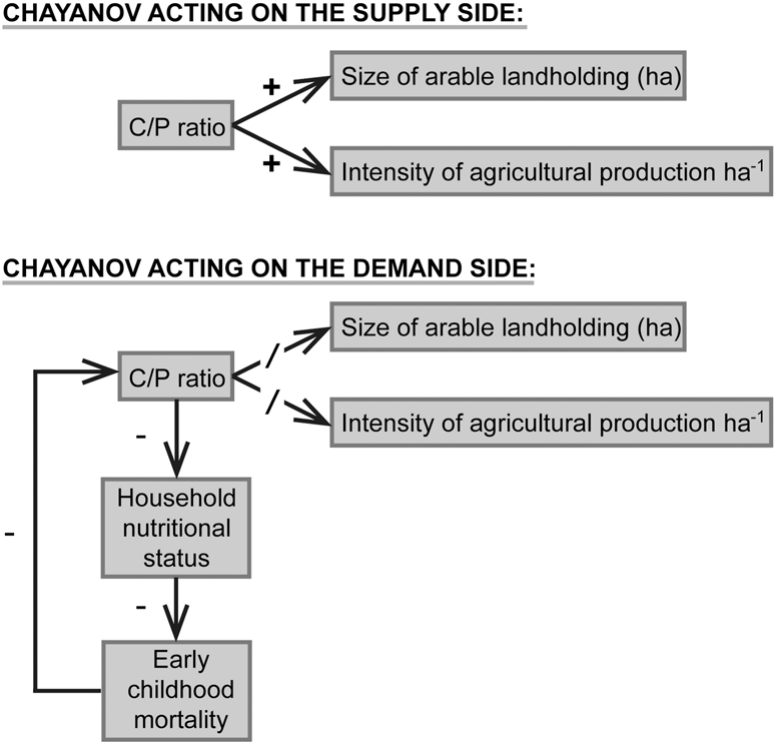
The conventional model that has C/P ratio acting purely on the supply side of the household economy (top) and an extended model that allows for possible effects on the demand side when supply-side responses are blocked or impeded (bottom). Pluses and minuses indicate the direction of the *bivariate* relationship between boxes.

Moreover, we argue on theoretical grounds that a strong, ever-present positive relationship between C/P ratios and agricultural intensity—a relationship that holds consistently across all preindustrial farming systems—is precisely what we should *not* expect to observe, even if Chayanov was fundamentally correct. There are two reasons to believe this. First, not all farming systems are equally susceptible to intensification or have the capacity for further intensification. In such cases, intensification is not always a workable option in the face of rising C/P ratios. Second, when further intensification is difficult or impossible, there are other potential outcomes resulting from high values of C/P, some of which may represent *failure* of the household farming enterprise. Fig. 1 illustrates our logic. We distinguish responses to high C/P ratios acting on the *supply* side (things that affect intensity of food production) from those that act on the *demand* side (things that influence household consumption). Chayanov’s own formulation of the model—and that of more recent researchers who have attempted to test it—looked exclusively at the supply side, as in the top part of Fig. 1. That is, they have examined the relationship between C/P ratios and either household landholding size or food yields per hectare of arable landholding. But in some circumstances *both* options may be unavailable (Fig. 1, bottom). More land may not be obtainable, it may be of poor quality, or the small household labor force may be unable to work it productively; greater yields may be unattainable because farm practices are, for all practical purposes, already as efficient and intensive as they can be or, perhaps, current practices cannot be intensified without resulting in environmental damage that would vitiate any resulting short-term increase in yield. In such circumstances, which we suggest may not be uncommon, things have to give way on the *demand* side: the nutritional status of household members will suffer and, as a result, the mortality rates of the most vulnerable individuals in the household, young children, will ultimately increase. Both responses—a decline in household nutritional status and an increase in early childhood mortality as C/P increases—have been hinted at in recent empirical studies [21–25]. Other demand-side responses are also possible, including emigration or fostering of one or more household members [26].

In addition to these demand-side pressures and possible poor nutritional outcomes, household composition may affect several of the socioeconomic determinants of child mortality. Following Mosley and Chen [27], household income and wealth, which are partially determined by household C/P ratios, influence access to food, water, household, clothing, fuel, transportation, medical and preventative care, and information. In addition, power relationships within the household affect access to resources, as does the educational status of mothers and fathers. The health and nutritional status of mothers is important to child survival, as much of their time is spent in childcare activities. Should there be competition between childcare activities and income-generating work for women, the survival chances of children may suffer if care is not of adequate quality or quantity. In other words, household composition, summarized by C/P ratios, can affect a number of important determinants of child health and survival.

In the present paper, we analyze field data from Na Savang (Lao PDR), an irrigated-rice growing village that has come close to exhausting the possibilities for further intensification using traditional farming practices and genetic resources. We ask whether increases in household C/P ratio have an adverse effect on the survival of children under the age of five within the household. In a later section on methods, we consider what additional variables need to be measured and controlled in order to obtain a valid picture of the relationship between C/P ratios and childhood mortality.

First, however, we confront one other measurement issue that has remained a major distraction for those interested in Chayanov’s model: how should we estimate C and P? Or, more precisely, how should we estimate the age- and sex-specific weights used to turn raw data on household membership into valid measures of C and P—the very things called “easily computed” by Netting [10]? Most weights used to date have been, at best, ad hoc and intuitive. Some authors have fallen back on the standard *dependency ratio* (usually defined as the ratio of household members ages < 15 and > 65 to those ages 15–65) despite the fact that it is crude and cross-culturally invalid (for a review, see [25]). We are aware of only one set of studies that has attempted to estimate the weights statistically from high-quality field data [28–30]. However, even these researchers’ complicated methods do not solve all the problems of estimating C and P. In our study, we refine and formalize a method originally suggested by Jennings [31] that we believe may avoid the problem: a sensitivity analysis of the effects of the *inevitable* mis-estimation of C and P on the study of early childhood deaths, described in detail below.

### The research site

**Fig 2:**
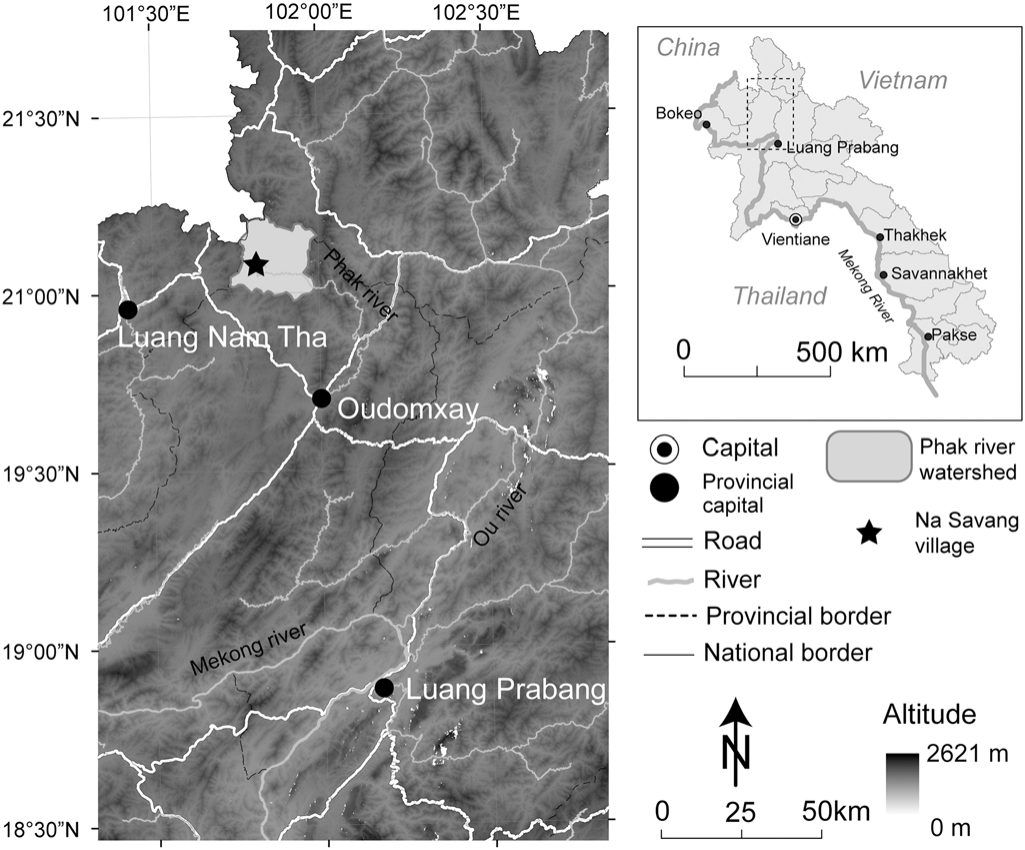
Location of the study site, Na Savang village, Oudomxay Province, northern Lao PDR.

Na Savang village (Namo District, Oudomxay Province, Lao PDR) sits at about 800 m above sea level in the lower reaches of the Phak River basin, a fairly isolated valley in a mountainous area of northern Laos (Fig. 2). The village is about 15 km from the Laos-China border crossing at Meo Chai and is beginning to feel economic influences from migrant Chinese—none of whom thus far live in the valley. There is, however, a more permanent Chinese presence in the surrounding region, and there is no question that the local Chinese influence is increasing. Many Chinese have moved to northern Laotian cities such as Oudomxay and Luang Nam Tha to open grocery shops, restaurants, and other businesses. In addition, local people such as the Lue, Hmong, and Akha have family connections in southern Yunnan and, at least in the recent past, visited that area frequently. Many of the inhabitants of Na Savang have affinal ties to the Lue and, through them, connections with Yunnan.

As of January 2006, the village population was 726, distributed into 137 households. Most residents of the village are ethnic Yang, who speak one of the Tai-Kadai languages of the Austro-Thai language family [32:177]. Before the *Pathet Lao* revolution of 1975, Na Savang village was the center of a small political unit called *Muang Ay*, which encompassed several villages made up of different ethnic groups including the Yang, Tai Lue, Khmu, Bit, Hmong, Lanten, Yao, Phousang, Phounyot, Kongsat, and Akha [33–35]. Na Savang, its valley, and its dependent villages still enjoy a degree of political and economic autonomy, although geographical barriers against the outside world are fast disappearing as more and better roads are built in the area.

Smallholder farmers in Na Savang make their livelihoods by pursuing a wide diversity of economic activities, mostly involving food production for both subsistence and, increasingly, cash sale. Small-scale irrigated farming of glutinous rice (and some non-glutinous varieties) has long formed the primary basis for subsistence, providing an estimated 85 percent of the caloric content of the local diet, with the remainder from a diverse set of activities. These activities include house gardens, dry-season vegetable cultivation in “fallow” paddy fields, vegetable gardening on the bunds (dikes) separating paddy fields, flood-recession farming along the banks of the Phak River, cultivation of cash crops (green vegetables, tobacco, bananas), livestock raising (mostly water buffalo, pigs, cattle, chickens, and ducks, along with a few turkey), fish farming in specially-built ponds, hunting, gathering of non-timber forest products, and trading with nearby villages, with Lao traders from Oudomxay, and with the occasional Han or Lue trader from China. Cash cropping, mainly for transient Chinese buyers, has been an important source of household income since 2003.

**Fig 3:**
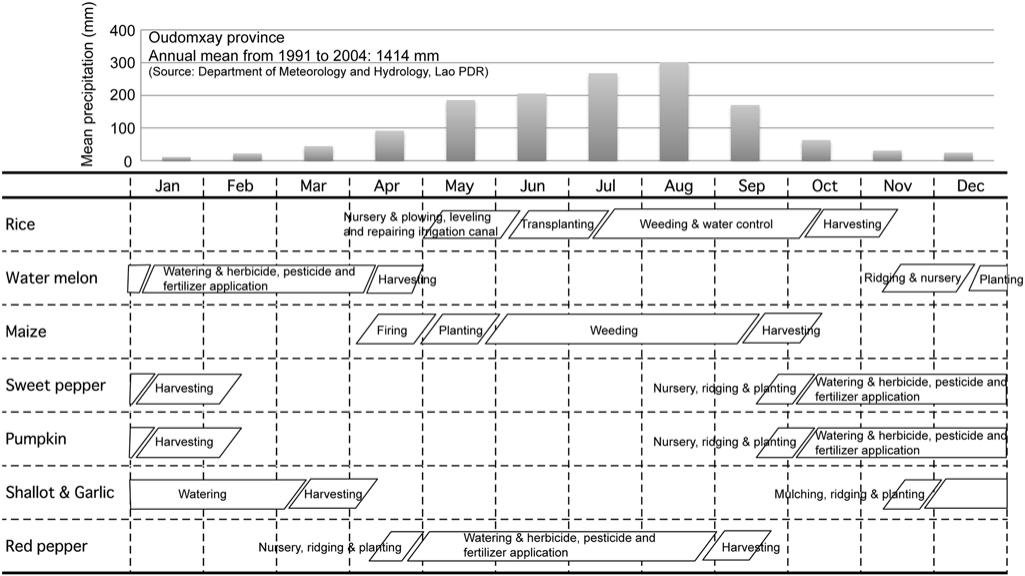
Seasonal patterns of rainfall (top) and major farming tasks (bottom), Na Savang village, northern Lao PDR.

The village as a whole has about 180 ha of rice fields, 99 percent of which is irrigated. Rice farming has had profound effects on local society and the yearly cycle of village life. The scheduling of labor in irrigated-rice farming is largely dictated by rainfall, whose sharp seasonal swings divide the year into labor-intensive and non-intensive periods (Fig. 3). During the non-intensive season people can devote ample time to the cultivation of crops other than rice, during the intensive season they cannot. As shown in Fig. 3, for example, the timing of maize production overlaps broadly with the rice-growing season. Consequently, the villagers of Na Savang can grow maize only by shoehorning its cultivation in between the most intensive rice-growing tasks (uprooting and transplanting seedlings, harvesting, threshing and winnowing); alternatively they can obtain maize by trade from people living at higher elevations who grow it in their swiddens. Livestock rearing does not compete significantly with rice farming because most animals are free-ranging—or were until the late 1990s when government health workers encouraged villagers to pen their pigs for reasons of hygiene.

**Fig 4:**
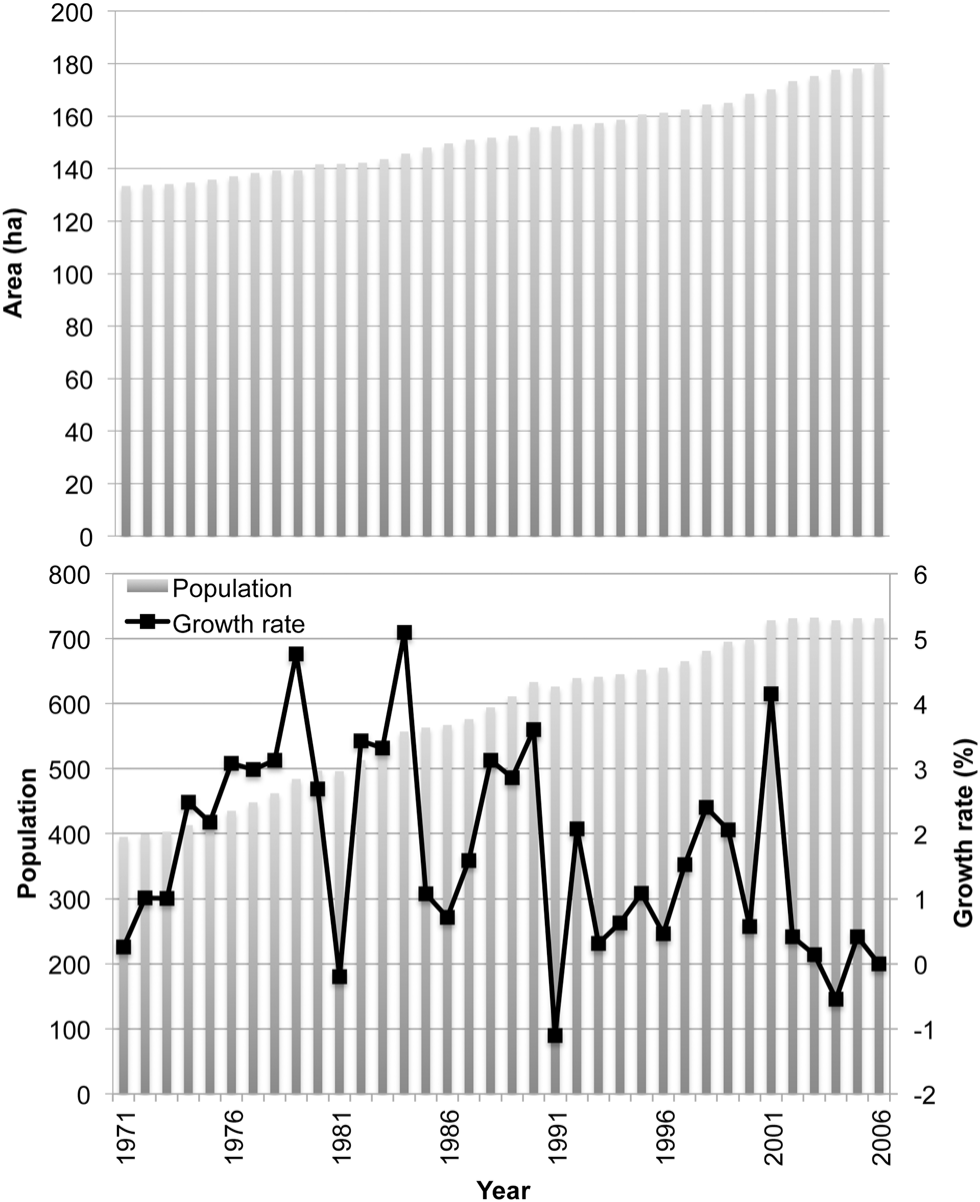
Growth in the total area of irrigated rice fields (top) and in village population (bottom) in Na Savang 1955–2006.

Lowland paddy fields are by far the most important source of subsistence for the village. Between 1960 and 2005, the area of irrigated-rice cultivation gradually increased from 120 to 177 ha [36–37], a process that coincided with the sustained growth of the village population (Fig. 4). The mean area of paddy per head of village population was 0.40 ha in 1960 and 0.25 ha in 2005, indicating that extension of wet-rice fields did not keep pace with demographic expansion—so that more and more people were living on less rice land per capita. Both the rate of population growth and the rate of creation of new rice fields have tapered off in recent years (Fig. 4), suggesting that this approach to intensifying food production may have run its course. Indeed, the valley floor is now about as full of irrigated rice fields as it reasonably can be. Still, recent yields seem to have been enough to allow most villagers to produce sufficient rice for household consumption [36],[38].

Informants claim they have never experienced serious rice shortages *except* during a period of government-imposed collective farming between the years 1979 and 1986. Although the Lao People’s Revolutionary Party promoted collectivization in all the wet-rice-growing regions of the country starting in 1978, it decided to suspend collectivization in most areas in 1979 because of large deficits in rice production at the national level [39–40]. The government’s retention of collective farming for such a long period in Na Savang may have been designed to undermine traditional forms of political organization in order to hasten incorporation of this region into the national polity and to support a military presence close to the Chinese border [36]. Collective farming in Na Savang continued until the mid-1980s despite the fact that villagers suffered from frequent rice shortages as a result. The amount of stored rice fell short of subsistence needs for 1–3 months out of every year during the period of collectivization, and villagers from Na Savang were forced to go to the nearby forested hillsides in the evening after finishing work in their paddy fields in order to do extra work in shifting cultivation (which was not subject to collectivization) and to exchange their pigs for rice in the mountain villages [36]. As shown below, these ad hoc remedies were not enough, and early childhood survival declined during collectivization.

## Materials and Methods

Fieldwork in Na Savang was conducted by the first author (S.T.) over a total of 18 months between 2004 and 2007. Additional data collection has continued intermittently up to the summer of 2013. As described in this section, data on demography and land use were collected for all 137 households in the village and used to reconstruct the dynamics of household structure and land ownership.

In both Laos and Japan, Institutional Review Board approval is not required or given for anthropological or agricultural fieldwork unless that fieldwork has an explicit medical component. Outside of medical schools there are no ethics committees, and therefore no such committee was consulted for this research. The villagers were informed about the types of data that would be collected and the resulting papers that would be written from those data. Verbal consent was given by the villagers but no written documentation was collected. The villagers were uniformly enthusiastic about participating in this research.

### Reconstructing population change

Village census records were lost in a series of fires, and there are no other prospectively-collected documentary sources that can substitute for them. Therefore, we conducted retrospective demographic interviews with all married villagers, asking them about their parents, siblings, and children, including each child’s name, sex, place of birth, year of birth, and year of death (if applicable), as well as about the couple’s year of marriage, number of marriages, houses where they lived after marriage, and the years when they moved from one domicile to another. These data were then cross-checked for internal consistency against overlapping data collected from close relatives, and when necessary the data were corrected through re-interview. In addition, we conferred with an elderly man and woman in the village who are renowned for their knowledge about local genealogies. Despite our care, the resulting information is subject to all the normal limitations of retrospective data, especially genealogical data [41–42]. It is likely, for example, that the further back in time we tried to go the more liable the data were to under-enumeration, recall error, and under-representation of families no longer living in the village because they either died out or emigrated. (For more recent decades, including the period covered by the current analyses, most emigrants have gone to Luang Nam Tha, Oudomxay, or Vientiane where most of them have been located and interviewed. Special pains were also taken to probe village genealogists for families that had died out locally. We believe that these forms of under-reporting or loss-to-follow-up have a negligible impact on the data used in this paper.) The data covering 1971 to 2006 were deemed to be reliable and complete enough to use in the analyses reported here.

### Defining households

We reconstructed households from 1971 to 2006 based on the local principle of *Kin nam kan*, *het nam kan* (Eat together, work together). There is a Lao term for household (*langkha huan*) that theoretically indicates a group of people living under the same roof. But while a listing of *langkha huan* would correspond closely to the *number* of extant houses, it would not always capture the relevant economic situation or residential pattern. Some newly-married couples build their own small house close to their parents’ house and move into it only after residing with the parents for up to seven years. The concept of *langkha huan* would count these as two separate households, even though the newly-weds are not yet economically independent of their parents—they often work together at farming and share provisions. After the parents divide and pass on paddy fields to their adult children, those children begin to work, eat, and live separately from their parents, although there are still many occasions for the larger group to cooperate economically. In some cases, however, parents pass on paddy fields to unmarried children who have not yet built a separate house and thus continue to reside with their parents. To reflect the reality on the ground, we decided to use the inheritance of paddy fields and whether the offspring have built their own house as the two essential criteria for the establishment of a new household.

Household landholdings were also measured and their geolocations were recorded into a geographic information system that relates families, households, and rice paddies.

### Statistical analyses

The outcome variable of interest in our analyses is the risk of death among children before five years of age, and the principal predictor variable that we want to assess is the C/P ratio of the household in which each child resided. If death occurred before age five, the outcome consisted of the age at death to the nearest year; otherwise, the observation was right-censored when the child turned five. (Since demographic events are known only to the nearest year, notional “birthdays” are assigned to the midpoint of the year.) Deaths to children after age five were thus omitted from the analysis, although their lifetimes before age five were included in the exposure term used to compute the mortality risks. The C/P ratio was allowed to vary over time as the household’s demographic composition changed up to (but not beyond) the date of the child’s death or censoring. The fact that the C/P-ratio effect ends at the child’s death avoids the potential problem that the death in question will itself affect the value of C/P, thus creating a spurious association between the two. Children could also be lost to follow-up through emigration of their families so any children they had who were under five at the time of a move were right-censored at the date of emigration.

We analyzed the risk of death using a mixed effects multivariate piecewise logit hazard model [43] that included C/P ratio as a predictor along with several other household-level control variables and several period-specific variables designed to control for changes in the village environment between 1971 and 2006 (see below). This model specification has several advantages: it does not require us to provide a parametric equation for the underlying hazard of death, it handles all forms of censoring that the data are subject to, it allows us to adjust for unobserved inter-household variation, and, despite its simple form, it is effectively age-specific since it allows us to enter the child’s age as a non-linear set of covariates. All variables other than sex of the child and mother’s age at the birth of the child were allowed to vary over time as described above for the C/P ratio. For these time-varying covariates, the entire history of values leading up to the death of the child or right-censoring (whichever occurred first) potentially has prospective predictive value. The model contains no lagged effects of time-varying covariates; rather, all effects are “instantaneous” in that they occur within the same year. In our preferred version of the model, we included a random-effect intercept term to adjust for unobserved heterogeneity among households and to correct for the non-independence of multiple deaths within the same household. In this treatment, intercepts are assumed to be normally distributed among households, with a mean and variance estimated from the data.

Using the estimated regression coefficient ${\hat{\beta }}_{j}$ from the logit model, we compute the effect size of the *j*-th covariate as$e^{{\hat{\beta }}_{j}}$. This quantity is the odds ratio measuring the proportional change in the odds of an early childhood death (adjusted for the confounding/control variables included in the model) that is expected to result from a unit increase in the value of the *j*-th covariate—assuming, of course, that ${\hat{\beta }}_{j}$ is correct. The uncertainty in the estimated effect size can be expressed by forming an approximate confidence interval for $e^{{\hat{\beta }}_{j}}$ by exponentiating the lower and upper confidence limits of ${\hat{\beta }}_{j}$ itself.

We have given careful consideration to the question of what household- and community-level variables need to be included in the analysis as statistical controls. We want to estimate the potential effect of household C/P ratio on early childhood mortality. But both C/P ratios and childhood mortality are closely associated with several other features of household demography and economy, and we need to adjust for the confounding influences of those features if we are to isolate the true effect of C/P *per se*. The age of the child in question affects both its risk of death and the household’s C/P ratio (as the child grows older its nutritional needs change, and it may even begin contributing to household production, as unlikely as that may be for a child less than five years old). The effect of age on the risk of early childhood death is known from a multitude of studies to be non-linear, so we have included both age and age^2^ as control variables. Age and age^2^ are obviously time-varying. (Age and age^2^ are also structurally colinear, a “problem” we might have solved by centering the observed ages. But in fact we’re not interested in the effect of age in its own right but only in controlling that effect statistically. The fact that age and age^2^ are confounded *with each other* is not pertinent to achieving that goal, and it would have been more difficult to evaluate how well we’ve controlled for the overall age effect if we had opted to center ages.) In most demographic and epidemiological analyses, the sex of the child is also an important predictor of the risk of death; it could conceivably affect the C/P ratio if it influenced the length of the subsequent birth interval as the family tries to achieve a desired ratio of male to female children. We therefore include sex as a control variable. Mother’s age at the birth of the child is another time-invariant covariate that often influences a child’s risk of death—the survival prospects of the offspring of older mothers in the rural developing world tend to be somewhat better than those of younger mothers [44–45]—and that is likely to be correlated with the C/P ratio. The size of the household (i.e. the number of members) is associated in a complex way with its C/P ratio; it may also reflect the level of intra-household competition for food resources and thus exert an independent effect on childhood mortality [25]. Household wealth differentials, if they exist, are also important potential confounders: a wealthy household may be better able to support more children and thus be less sensitive to an increase in C/P. Wealth differentials, while subtle, certainly exist in Na Savang and are perhaps most clearly reflected in the quality of the materials used in house construction (traditional bush materials versus concrete floors and walls and corrugated-iron or tile roofing). Unfortunately the current data-set does not include information on building materials, so we have opted to use landholding size in hectares as a proxy measure of household wealth. Household size and landholding size both vary over time. In preliminary analyses we included a C/P × landholding interaction term, but its effect size was indistinguishable from one (no effect) and it did not change any of the other covariate estimates substantially. In the interests of parsimony, we left the C/P × landholding interaction out of our final model. Another alternative model specification examined the effects of C and P separately as the weighted number of consumers or producers might affect child survival independently. However, the estimated coefficients for C and P were not statistically significant. The results for these and other alternative models can be obtained from the authors on request.

In addition to these household-level covariates, the community-wide history of Na Savang over the study period 1971–2006 needs to be taken into account. We have therefore included several chronological control variables in the analysis (such variables are by their nature time-varying). Year of observation can serve as a proxy for all the changes that occurred in Na Savang between 1971 and 2006 that we have not explicitly recorded. We split the observation period into (roughly) decadal intervals: 1971–1980, 1981–1990, 1991–2000, 2001–2006. Note that the last period is shorter than the others, which should be borne in mind when interpreting the associated coefficients; again, because we included period strictly for the purpose of statistical control, this discrepancy is of no substantive importance in the current analysis.

In addition to the generic effects of period, there are other period-specific events and processes that are recorded separately in our database by year of occurrence. These include natural and man-made disasters (floods, droughts, fires) and government-imposed collective farming (1979–1986). All these period-specific effects are modeled as dummy variables, and interactions among them were included in some versions of the model (results available on request). But when we tried to include the chronological interactions in the model with the random household intercept, it failed to converge. In our opinion, controlling for unobserved inter-household heterogeneity was far more consequential for interpreting our results than controlling for interactions among chronological variables, all of which interactions were small in effect and had large *p* values. Inclusion of the interaction terms had only minor effects on the regression coefficients for any of the main effects, except (unsurprisingly) for some of the chronological controls. In this paper we present the results only for the more parsimonious model with a random household intercept.

**Fig 5:**
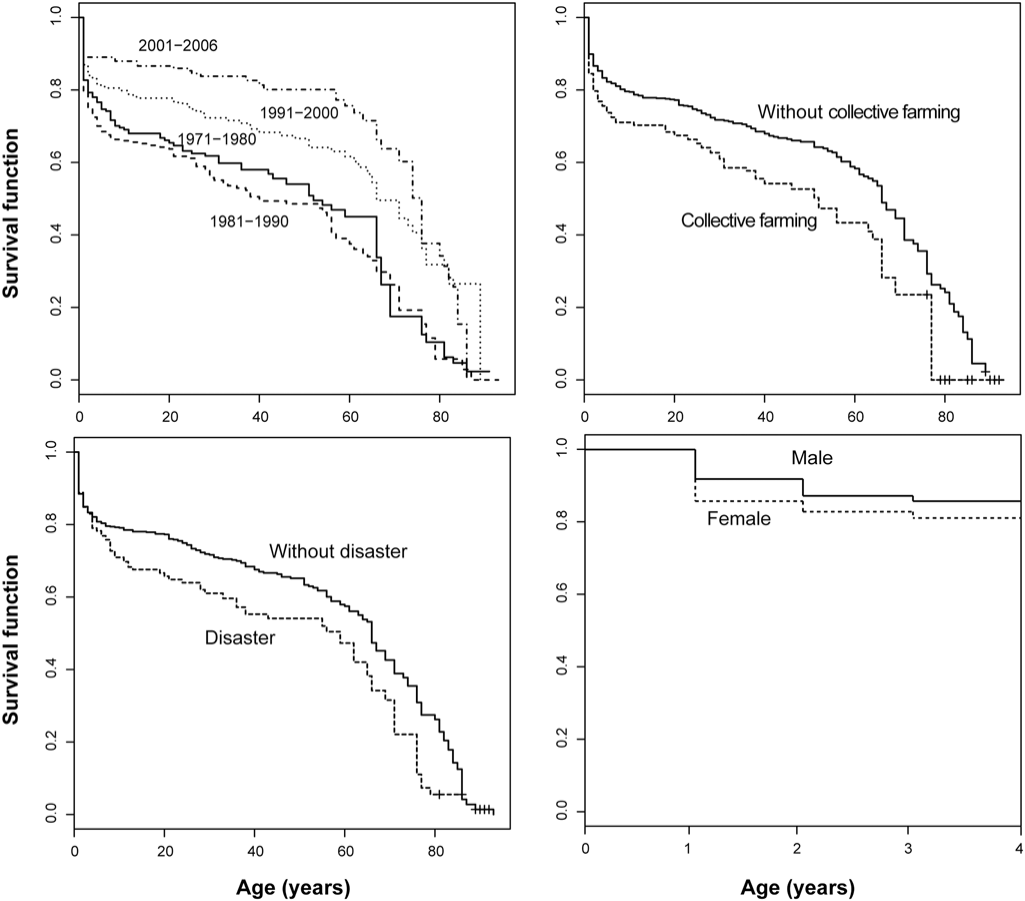
Kaplan-Meier (product-limit) estimates of the probability of surviving from birth to each subsequent age, Na Savang village (1971–2006), broken down by period (top left), whether collective farming was in force or not (top right), whether a disaster (flood or drought) occurred in a given year (bottom left), and the sex of the individual at risk (bottom right). Note that survival by sex is shown only for the first four years of life.

To provide a sense of how important some of our control variables are, we computed Kaplan-Meier estimates of their effects on survival in Na Savang (Fig. 5). From the survival functions by period, it seems clear that survival before age five (and at most other ages) improved markedly after about 1990, coinciding with the loosening of communist control over the economy. Similarly, the period of collective farming was associated with increased mortality before age ten compared to later periods after forced collectivization was rescinded. As one would expect, mortality was higher during years of environmental and man-made disaster, although the increase appears to have been greater for children aged five to ten than for infants and toddlers (a possible reflection of the protective effects of breastfeeding). More surprisingly, the child’s sex did not appear to have an appreciable effect on its risk of death before age four (females experienced very slightly higher mortality than males). Regardless, we considered sex an important control variable and included it in the multivariate logit model anyway.

### “Estimating” C and P

We have already alluded to the difficulty of estimating meaningful values of C and P from empirical data [28]. We have not tried to estimate C and P for Na Savang in any formal way (although we may try to do so in the future). Instead, we have adopted a simulation approach, first suggested by Jennings [31], to test the sensitivity of our model results to the inevitable errors contained in our C and P weights. We begin with an initial set of age- and sex-specific weights for consumption and production that seem plausible (and we would put it no more strongly than that) based on our experience in Na Savang (Table 2). (There was some debate among the co-authors about whether we may have set the consumption weights for older men and women too low; but if we did, that would result in *diminishing* the apparent effect of C/P ratio on childhood mortality, not biasing it upward.) We then perturb those weights at random to simulate estimation error. Using a pseudo-random number generator, we draw perturbation sizes (positive or negative) from a uniform distribution, chosen for its simplicity. A uniform distribution is based on two parameters, *a* and *b*, that specify the range of potential perturbations; for each age and sex (and for production and consumption separately), we selected values of *a* and *b* based on the inter-quartile ranges reported by Lee and Kramer [28:483] for their individual C and P values. We consider this approach conservative (i.e. to err in the direction of *over*-estimating the actual degree of error rather than under-estimating it) since the values of C and P reported by Lee and Kramer necessarily involve both estimation error and real differences among individuals. In addition, use of a uniform distribution places more weight on larger perturbations than would that of, say, a normal error distribution, and this also serves to over-estimate the amount of error.

**Table 2:** Notional (baseline) age- and sex-specific weights used to compute C and P for Na Savang households.

Age (years)	Production		Consumption	
	Male	Female	Male	Female
0–4	0.0	0.0	0.1	0.1
5–9	0.0	0.0	0.1	0.1
10–14	0.1	0.0	0.2	0.1
15–19	0.3	0.2	0.4	0.3
20–24	0.5	0.4	0.6	0.5
25–29	0.7	0.6	0.8	0.7
30–34	0.9	0.8	1.0	0.9
35–39	1.0	0.9	1.0	0.9
40–44	1.0	0.9	1.0	0.9
45–49	1.0	0.9	1.0	0.9
50–54	1.0	0.9	1.0	0.9
55–59	1.0	0.9	1.0	0.9
60–64	1.0	0.9	1.0	0.9
65–69	0.8	0.7	0.9	0.8
70–74	0.3	0.2	0.4	0.3
75–79	0.3	0.2	0.4	0.3
80–84	0.2	0.1	0.4	0.3
85+	0.0	0.0	0.4	0.3

All values are scaled to those of males ages 35–64 years and thus are unit-free.

We then used an iterative procedure that randomly generated weights (fixed initial value + random perturbation) for each age × sex group, substituted them in the logit hazard model along with all our control variables, computed and saved the model results, and then started the whole process again. The simulations were run 1000 times, resulting in 1000 independent, randomly-perturbed estimates of the regression coefficient β for the influence of household C/P ratios on child mortality. We then converted those β estimates into effect sizes (odds ratios) by exponentiating them, as described above. Plots of the resulting distribution of β coefficients and effect sizes (see below) provide a useful visual indication of the probable error introduced into our analysis owing to the mis-estimation of C and P.

## Results

Results for our preferred model are shown in Table 3 (results for alternative model specifications, which provided substantially similar estimates, can be obtained from the authors on request). The random intercept term, designed to correct for unobserved variation in household characteristics, yielded a variance that, while rather low (0.107), is still more than three times its standard error (0.029) above zero. As a consequence, we judged that it was important to include the random intercept in order to obtain a clearer picture of the influence of *observed* household demographic and economic variables, including C/P ratio, on early childhood mortality, which could be confounded by unobserved heterogeneity. The chronological controls, on the other hand, mostly had small effects. (The exception is the dummy variable for the period 1981–1990, which appears to increase the risk of childhood death almost three-fold. We suspect it is no coincidence that this period overlaps with the time of centrally-imposed collectivization of farming in 1979–1986. If that suspicion is correct, then this particular period effect is almost certainly confounded with the dummy variable for collectivization, which would obscure the reasons for the temporal trend but not affect our estimate of the influence of C/P.) Those effects were not changed in any important way by including interaction terms for chronology (results available on request). The other control variables, some of which had moderately large effect sizes, were generally in the direction expected (see the discussion below). In our opinion, these aspects add further validity to the selected model.

**Table 3:** Mixed-effect logit hazard model with random household-level intercept, early childhood mortality (ages < 5 years), Na Savang Village, northern Laos 1971–2006.

Predictor variable	Estimated *β* coefficient	Standard error	Effect size^a^	*z* value	Corrected^b^ *p*
***Demographic and household (HH) variables:***					
Child’s age	-1.182	0.222	0.307	-5.335	0.001
Child’s age^2^	0.172	0.062	1.187	2.766	0.018
Child’s sex (f = 1)	0.116	0.179	1.123	0.645	0.999
Mother’s age	-0.027	0.012	0.973	-2.206	0.081
HH landholding (ha)	0.000	< 0.001	1.000	-2.125	0.102
HH size	0.047	0.024	1.048	1.945	0.156
C/P ratio	2.199	0.865	9.020	2.541	0.032
Child’s age	-1.182	0.222	0.307	-5.335	0.001
Child’s age^2^	0.172	0.062	1.187	2.766	0.018
***Chronological control variables:***					
Collectivization? (y = 1)	0.059	0.252	1.060	0.233	0.999
Disaster? (y = 1)	0.050	0.256	1.051	0.194	0.999
Period 1 (1971–1980)	1.244	0.507	3.470	2.455	0.126
Period 2 (1981–1990)	1.048	0.520	2.851	2.014	0.132
Period 3 (1991–2000)	0.594	0.515	1.812	1.154	0.747
Period 4 (2001–2006)	reference category				
***Random household intercept:***					
Estimated mean = -5.317 ± 1.274					
Estimated variance = 0.107 ± 0.029					

^a^Effect size = exp(estimated β coefficient). No effect = 1.0.

^b^Bonferroni correction for multiple tests against alternative models discussed in text (results available on request).

*N* = 148 early childhood deaths among 3075 children at risk. Parameters of the random household intercept are presented as estimate ± one standard error.

Table 3 suggests a substantial adverse effect of rising C/P ratios on mortality before the age of five. The estimated β coefficient is 2.199 ± 0.865 (Bonferroni corrected *p* = 0.032), and the associated odds ratio is 9.02. Taking this effect size at face value, we would have to conclude that a unit rise in C/P increases a household’s early childhood mortality rate nine-fold or by 900 percent. This is, by any standards, an enormous effect, and we discuss its credibility below. For now, however, we note that a unit increase in C/P is itself an enormous change, spanning the entire range of cycling C/P values cited in the literature [28]. In other words, a unit increase in C/P is about as large an increase as you would expect to observe over a household’s entire demographic history.

We note the availability of public healthcare services and changes in women’s work rates on childhood mortality. Beginning in the late 1990s, the government and NGOs provided women and children’s healthcare and modern medicines. These services could dramatically improve the recipients’ health status. But C/P would restrict a household’s access to the services and consequently affect mortality. At the same time, women’s (and men’s) workloads seemed to lighten right after the end of collectivization, and the mortality rate was going down irrespective of the availability of the healthcare services. C/P also decreased right after the end of collectivization because households started to separate into nuclear or stem families. At any rate, C/P is able to capture the influence of household structure on mortality, although the causes of change in household structure are multitudinous.

**Fig 6:**
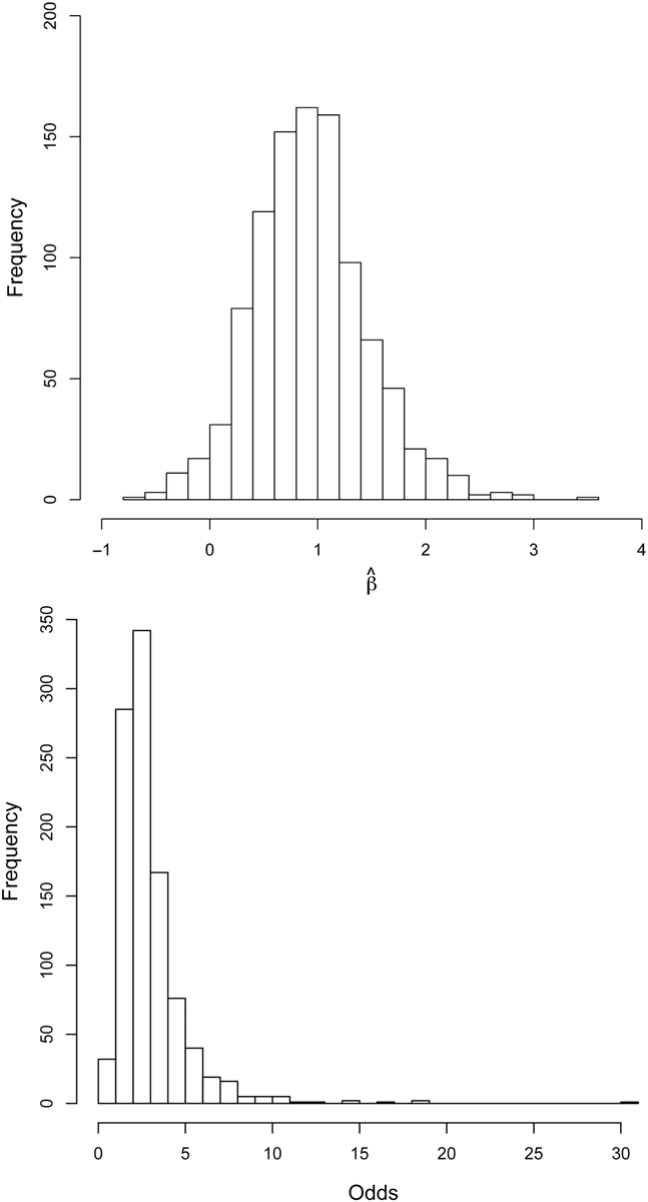
Distributions of simulated regression coefficients (top) and effect sizes (bottom) from the regression of early childhood mortality on C/P resulting from 1000 random perturbations to the age- and sex-specific weights used to compute C and P.

The sensitivity analyses we conducted to evaluate the likely effects of error in measuring C and P appear to support the conclusion that the adverse effect of an increase in C/P on early childhood mortality is real, even if it is not as large as the estimated effect size would suggest. Fig. 6 shows the simulated distributions of both the β coefficients (top) and odds ratios or effect sizes (bottom) for C/P induced by varying both C and P through a range of values suggested by Lee and Kramer’s [28] empirical results. While our estimates of β (2.2) and its odds ratio (9.0) for Na Savang decidedly fall in the upper tails of the simulated distributions, only 33 out of 1000 results (about three percent) are equal to or below zero for β and equal to or below 1.0 for the effect size. On this basis, we conclude that the adverse effect of increasing C/P ratios on childhood survival is likely to be real, even if we remain skeptical about its precise magnitude. In Na Savang, Chayanov operates on the demand side. Whether he also operates on the supply side is a question for future research.

## Discussion

Na Savang is a community that has pushed local agricultural intensity about as high as it can go using traditional practices and genetic resources. Much of the valley bottom—which includes all the land available to the village—is now covered with irrigated rice fields. The village has about 180 ha of rice fields, 99 percent of which is irrigated; this area represents approximately 65 percent of all the land to which Na Savang has use-rights, leaving limited space for other crops, animal grazing, house sites, or public spaces such as the local Buddhist temple or primary school, as well as limited space for the further expansion of irrigated paddy. The average extent of rice fields has been squeezed down to 0.25 ha per capita [37]. Nonetheless, virtually all households are deeply involved in irrigated-rice farming, mostly for their own subsistence. It is difficult to imagine, under these circumstances, that rice yields could be increased much by reclaiming more land, working harder, or applying traditional techniques more efficiently [38]. Yet the demographic fortunes of each household continue to change as it follows its inexorable life-cycle trajectory. If households in Na Savang can no longer offset a rise in food-consumption needs by intensifying rice production using traditional practices, or by enclosing more land, what are they to do? They may, of course, eventually adopt new non-native varieties of rice, mechanized methods of cultivation, inorganic fertilizers, and store-bought pesticides, but at the time our field data were collected such things had occurred only to a limited extent. (This is starting to change rapidly.) The only other recourse, then, is to cut back on the demand side of the household economy—that is, reduce food consumption, which results in lower nutritional statuses and increased risks of illness and death among the household’s most vulnerable members, its young children. Our analysis suggests that this unfortunate train of events may already be happening in Na Savang, where an increase in household C/P ratio appears to exert a substantial effect on the odds of an early childhood death.

The effect size of the C/P ratio estimated here, equivalent to about a nine-fold increase in the odds of an early childhood death for each unit increase in the household’s C/P ratio, is likely to be an overestimate. Regardless, a unit increase in C/P ratio is itself large, spanning more or less the entire range in that variable reported in other studies (by comparison, a unit increase in, say, household size or mother’s age is proportionately much smaller). Therefore, while we are skeptical about the estimated effect size, we are confident that it is not zero and that it is positive. Our simulation results support this conclusion. We acknowledge that the specific results reported in Table 3 hinge on the precise age- and sex-specific weights used in the analysis. This is true of every test of the Chayanovian model. These weights have not been convincingly estimated in previous research, not even through the efforts of Lee and Kramer [28], and they are certainly not convincingly estimated in the present paper. But, following the suggestion of Jennings [31], we have offered an alternative way to deal with the seemingly unavoidable uncertainty involved in estimating these weights: by conducting sensitivity analyses of the probable effect of errors in measuring C/P. What our sensitivity analyses show is that almost all simulated effect sizes fall above 1.0, and some are consistent with very large effects (Fig. 6).

If C/P really does have an important effect on early childhood mortality, how does it work? In other words, what do we think are the likely mechanisms linking the household’s consumption needs (relative to its work capacity) to the risk of death among its youngest members? Our answer for Na Savang must be largely speculative, at least for the time being, but one point seems worth making by way of hypothesis. We do not think children die when C/P is high because they starve to death in any literal sense of that term, except perhaps in a very few extreme cases. Instead, we believe that more subtle nutritional changes are at work. We also suspect that those subtler changes are influencing early mortality primarily via their effects on the child’s immune system—that is, on its ability to mount an effective immune response against infectious diseases, known to be the major causes of death among children in the rural developing world [46]. The immunosuppressive effects of under-nutrition, even mild under-nutrition, are well-documented [47–49]. If this mechanism is indeed a major linkage between C/P and childhood mortality, then it has an important implication for future analyses: we will need to control statistically for breastfeeding, which has both nutritional and immuno-protective value for the nursing child that might help ameliorate any adverse effect of household composition [50]. In general, if we were to combine the sorts of data examined in the present paper with good evidence on cause of death, child’s (and perhaps mother’s) nutritional status, breastfeeding behavior, and the child’s immunocompetence, which can now be roughly measured in the field [51–52], it would greatly clarify what it is that C/P is actually doing.

In the final analysis, C/P is a complicated theoretical construct. Just to think about P, household productive capacity includes (among other things) components related to physiological work capacity, the nutritional status of workers, the energy efficiency of available tools, and the division of labor by age and sex, the last two of which are at least partly cultural in nature. C must be just as complicated. Neither C nor P is a unitary, single-dimensioned variable—and the C/P ratio is even further from being one. Perhaps the ultimate solution for the problem of measuring C/P is to dissect C and P into their many parts and then measure them separately. Such a task will require good, comprehensive models of the determinants of both C and P, and probably some new measurement techniques. It will still, no doubt, involve substantial measurement error, but it might be possible to characterize that error empirically rather than through computer simulations.

As a final point, we note that we attempted to identify important confounding influences that need to be controlled in order to unmask any effect of C/P ratios. Failure to do this has been one of the most serious shortcomings of earlier tests of Chayanov’s model. The effects estimated for these confounding variables are, for the most part, plausible and in the direction expected from other studies. For example, the two controls for child’s age together nicely capture the usual decelerating decline in mortality observed in very young children, and the modest but ameliorating effect of mother’s age is consistent with findings from past research [44]. Household size has a tiny effect, but one that is in the expected direction; the small effect of landholding (“wealth”) probably reflects limited heterogeneity in that variable among villagers. The period of forced collectivization increased early child mortality by about 300 percent, natural disasters increased it by perhaps six percent, more recent trends are generally toward declining risk of death—and so forth. Only the sex of the child seems to defy expectation, but we note that this lack of a clear-cut sex difference has in fact been found in other parts of mainland Southeast Asia [53]. Moreover, according to elderly women in Na Savang, there has never been a difference in mortality by the sex of the child (our results are consistent with the villagers’ perception). We note, however, that Na Savang practices bilateral descent: they are primarily patrilocal or neolocal after a few to several years of matrilocal residence. While it is unclear from the present analysis whether the kinship system has some effect on the village’s mortality, it is worth examining this issue in our future work. Interestingly, estimation of the random household intercept made it appear that some, but not much, variation does exist among households in what may be a wide array of unrecorded variables: the estimate for the variance (0.107) is small but its standard error (0.029) is even smaller. We admit that viewing the effect of C/P through all these confounding influences is not straightforward, but the test presented here suggests that Chayanov was right, even if in a somewhat more complicated way than he originally thought.

## Supporting Information

S1 File (CSV)
